# SARS-CoV-2 variants Alpha, Beta, Delta and Omicron show a slower host cell interferon response compared to an early pandemic variant

**DOI:** 10.3389/fimmu.2022.1016108

**Published:** 2022-09-30

**Authors:** Larissa Laine, Marika Skön, Elina Väisänen, Ilkka Julkunen, Pamela Österlund

**Affiliations:** ^1^ Expert Microbiology Unit, Department of Health Security, Finnish Institute for Health and Welfare, Helsinki, Finland; ^2^ Infection and Immunity, Institute of Biomedicine, University of Turku, Turku, Finland

**Keywords:** SARS-CoV-2, Omicron, variants, mutations, replication, innate immunity, interferon

## Abstract

Since the start of the pandemic at the end of 2019, arising mutations in SARS-CoV-2 have improved its transmission and ability to circumvent the immunity induced by vaccination and previous COVID-19 infection. Studies on the effects of SARS-CoV-2 genomic mutations on replication and innate immunity will give us valuable insight into the evolution of the virus which can aid in further development of vaccines and new treatment modalities. Here we systematically analyzed the kinetics of virus replication, innate immune activation, and host cell antiviral response patterns in Alpha, Beta, Delta, Kappa, Omicron and two early pandemic SARS-CoV-2 variant-infected human lung epithelial Calu-3 cells. We observed overall comparable replication patterns for these variants with modest variations. Particularly, the sublineages of Omicron BA.1, BA.2 and a recombinant sublineage, XJ, all showed attenuated replication in Calu-3 cells compared to Alpha and Delta. Furthermore, there was relatively weak activation of primary innate immune signaling pathways, however, all variants produced enough interferons to induce the activation of STAT2 and production of interferon stimulated genes (ISGs). While interferon mRNA expression and STAT2 activation correlated with cellular viral RNA levels, ISG production did not. Although clear cut effects of specific SARS-CoV-2 genomic mutations could not be concluded, the variants of concern, including Omicron, showed a lower replication efficiency and a slower interferon response compared to an early pandemic variant in the study.

## Introduction

Severe acute respiratory syndrome coronavirus 2 (SARS-CoV-2) is the causative agent of COVID-19 of which there has been over 570 million cases and 6.4 million deaths as of August 2022 (1). During these past two years, the virus has evolved into hundreds of variants which have affected the outcome of the pandemic. In Spring 2020, a D614G mutation in spike (S) protein and a concurrent P232L mutation in the RNA-dependent RNA polymerase (RdRp) made the virus more infectious and transmissible and variants with these mutations overran the original Wuhan SARS-CoV-2 strain (2–5). Mutations continued to accumulate and by the end of 2020, the variants of concern (VOCs), Alpha and Beta, had emerged and started to spread throughout the world (6). The Alpha variant became the globally dominant virus in May 2021 until it was substituted by the Delta variant in the end of summer 2021 (7). Thereafter, at the end of 2021 a genetically new variant, Omicron, swept across the world at an unprecedented speed (8). As herd immunity to SARS-CoV-2 induced by vaccination and infection rises, we have to be on alert for virus evolution. To further understand how the virus mutations contribute to pathogenicity or transmissibility of the variants, we need to study their ability to replicate and induce host cell responses in human cells.

SARS-CoV-2 belongs to the *Sarbecovirus* sub-genus of *Betacoronaviruses* and it has a large, single-stranded, positive-sense genome with approximately 30 000 bases (9). Starting from the 5’ end, two thirds of the genome comprise the open reading frames ORF1a and ORF1b, which are translated into polyproteins that are post-translationally cleaved into 16 non-structural proteins (NSPs). These form the viral replication and transcription complex (RTC) that drives viral genomic and sub-genomic RNA (gRNA, sgRNA) replication (10). The viral structural (spike (S), envelope (E), membrane (M), nucleocapsid (N) proteins) and accessory proteins (11) reside in the 3’ end of the genome. The SARS-CoV-2 entry into the host cells is initiated by the attachment of the S protein receptor-binding domain (RBD) to the angiotensin-converting enzyme 2 (ACE2) receptor (12). Fusion of the viral and host membranes occurs *via* cleavage of the S protein multi-basic cleavage site (MBCS) at the boundary of its two domains, S1 and S2, by furin which is followed by further priming at a S2’ site by a transmembrane serine protease 2 (TMPRSS2) (13–15). Entry of SARS-CoV-2 may also occur *via* the endosomal entry route where cleavage of S by Cathepsin L facilitates fusion of viral and endosomal membranes (16, 17). The genomic RNA is released into the host cell, the RTC is formed and the virus creates a replication organelle where gRNA and sgRNA replication occurs (18). SgRNAs are translated by host cell ribosomes to synthesize the structural and accessory proteins (10), the new viral membrane is formed, genomic RNA is packaged with N protein and new virions are assembled (19, 20).

Airway epithelial cells function as the first defense mechanisms against respiratory pathogens as they form a complex protective environment that includes physical barriers, mucociliary clearance and production of surfactants which bind pathogen-associated molecular patterns (PAMPs) (21). Lung epithelial cells also express several pattern recognition receptors (PRRs) on the outer membrane, endosomes and in the cytoplasm, which activate innate immune responses that limit infection and enhance clearance of the pathogen. SARS-CoV-2 RNA replication products have been shown to be recognized mainly *via* the cytoplasmic sensors MDA5 and RIG-I, while of the Toll-like receptors (TLRs), only TLR2 on the outer cell membrane has been clearly shown to be involved in SARS-CoV-2 mediated immune signaling (22). SARS-CoV-2 infection activates the signaling cascades that induce the expression of type I and III interferons (IFNs) and further the IFN signaling pathway (22, 23). Several coronavirus proteins antagonize the signaling events inhibiting the activation of the immune response (23).

Comparative analyses how different SARS-CoV-2 variants replicate and activate innate immunity are lacking. Thorne et al. have compared innate immune activation of Alpha and wild type variants (24) and immune activation of early pandemic variants have been studied (25, 26). Here we analysed the ability of six genetically different pre-Omicron SARS-CoV-2 isolates to replicate and induce innate antiviral immunity in a human lung epithelial cell model system. Our study included two early pandemic variants (Fin3 and Fin22), the VOCs Alpha (Fin34-α), Beta (Fin32-β) and Delta (Fin37-δ) as well as a Kappa (Fin40-κ) variant. Furthermore, the replication characteristics and host cell interferon induction of three Omicron sublineages (Fin55-BA.1, Fin58-BA.2 and recombinant Fin60-XJ) were compared to Alpha and Delta. We showed that the replication profiles of the pre-Omicron variants in the human lung epithelial cell line, Calu-3, follow a similar trend, with only modest differences. However, the replication of all Omicron sublineages was attenuated, especially that of BA.2. We detected low levels of primary innate immune signaling but sufficient interferon induction for a robust activation of the JAK-STAT pathway. Interferon mRNA expression correlated with the intracellular levels of viral RNAs, but all the variants induced similar production levels of ISGs. Interferon and cytokine production was induced at a slow pace in all but one early variant (Fin22), which interferon response peaked 24 h earlier. These results suggest that the effects of the SARS-CoV-2 mutations are very complex as various mutational profiles resulted in similar replication and host cell activation patterns. However, looking at the overall picture from early pandemic SARS-CoV-2 variants and VOCs to Omicron, the shift to lower replication efficiency and slower interferon induction suggests that accumulated mutations likely contribute to viral adaptation.

## Materials and methods

### Cell culture

VeroE6-TMPRSS2-H10 cell line (27) and a human airway epithelial cell line, Calu-3 (ATCC, HTB-55) were maintained in Eagle’s minimum essential medium (E-MEM) supplemented with 0.6 µg/mL penicillin, 60 µg/mL streptomycin, 2 mM L-glutamine, 20 mM HEPES. The cell growth medium contained 10% and 15% fetal bovine serum (FCS, Sigma Aldrich) for VeroE6-TMPRSS2-H10 and Calu-3, respectively. Cells were incubated at 37°C in 5% CO_2_.

### Generation of virus stocks

SARS-CoV-2 variants used in the present study include Fin3 (B.1.1.29, hCoV-19/Finland/FIN-3/2020, EPI_ISL_2365908, ON531991), Fin22 (B.1.258, hCoV-19/Finland/THL-202039825/2020, EPI_ISL_3471857, ON532015), Fin32-β (B.1.351, Beta variant, hCoV-19/Finland/THL-202101018/2021, EPI_ISL_3471851, ON532063), Fin34-α (B.1.1.7, Alpha variant, hCoV-19/Finland/THL-202102301/2021, EPI_ISL_2590786, ON532062), Fin37-δ (B.1.617.2, Delta variant, hCoV-19/Finland/THL-202117309/2021, EPI_ISL_2557176, ON532078), Fin40-κ (B.1.617.1, Kappa variant, hCoV-19/Finland/THL-202109869/2021, EPI_ISL_2506747, ON532082), Fin55-BA.1 (Omicron sublineage BA.1, hCoV-19/Finland/THL-202126660/2021, EPI_ISL_8768822, ON532087), Fin58-BA.2 (Omicron sublineage BA.2, hCoV-19/Finland/THL-202203911/2022, EPI_ISL_9695067, ON532088), Fin60-XJ (Omicron sublineage XJ, hCoV-19/Finland/THL-202205928/2022, EPI_ISL_10148532, ON532089). The viruses were isolated from COVID-19 patient nasopharyngeal swab samples and the sequences had been confirmed to be the above SARS-CoV-2 variants by next generation sequencing. Virus isolation was carried out in VeroE6-TMPRSS2-H10 cells followed by a second passage in a fresh culture of the same cells to generate virus stocks. Virus stocks were collected two to five days after seeding the second passage. Fin58-BA.2 and Fin60-XJ required a third passage in VeroE6-TMPRSS2 cells for sufficient replication to generate usable viral stocks. Virus titers (TCID_50_/mL) were obtained by endpoint dilution in VeroE6-TMPRSS2-H10 cells and they were as follows: Fin3 (1x10^8^), Fin22 (1x10^7^), Fin32-β (1x10^8^), Fin34-α (1x10^7^), Fin37-δ (1x10^7^), Fin40-κ (1x10^8^), Fin55-BA.1 (1x10^7^), Fin58-BA.2 (1x10^7^) and Fin60-XJ (1x10^7^). All virus stocks were also whole genome sequenced. All work with infectious SARS-CoV-2 virus was carried out in the biosafety level 3 facility at the Finnish Institute for Health and Welfare, Finland.

### Infectivity assay

A total of 1 x 10^6^ Calu-3 cells were cultured in 12-well plates and after three days confluent cells were infected with SARS-CoV-2 variants. Briefly, viruses were added to the cells at MOI 1 based on the titers determined by the endpoint dilution assay. The dilution of the stock viruses for the infection were done in E-MEM media supplemented as above but without FCS. Cells were incubated with the inoculum viruses for 1h at 37°C in 5% CO_2_ after which the medium containing virus was removed, the cells were washed once with PBS and supplemented with E-MEM containing 2% FCS. The 1h sample of the supernatant, cellular RNA and cell lysate for protein analysis were collected at this point. Cells were then placed in the incubator at 37°C in 5% CO_2_ and subsequent samples were collected at 6, 24, 48 and 72 hours post-infection (p.i.).

### Endpoint dilution assay

VeroE6-TMPRSS2-H10 cells were seeded into 96-well plates 24 hours prior to infection. Serial dilutions of the stock viruses or the supernatants collected from the infectivity assay at different time points were made and eight replicate wells were inoculated with each sample dilution. Cytopathic effect (CPE) was observed at three or six days p.i. to determine whether a well was positive or negative for viral growth and the virus titers represented as TCID_50_/mL were calculated using the Spearman-Kärber method.

### Sendai infection of Calu-3

Sendai virus (SV) (strain Cantell) was propagated in 11-day-old embryonated chicken eggs at 36°C for 3 days and stock virus titer was determined as infectious units/ml in human primary dendritic cells (DCs), and it was 1x10^9^ pfu/ml. A total of 1 x 10^6^ Calu-3 cells were infected with SV at MOI of 5 based on the titers determined in DCs. Calu-3 cells were collected at 8 h p.i. for immunoblotting.

### Isolation of RNA and RT-qPCR

Total cellular RNA and cell culture supernatant viral RNA (vRNA) were isolated using the RNeasy mini kit (Qiagen). For cellular RNA, DNase digestion (RNase-free DNase kit, Qiagen) was included, and the total RNA concentration was measured using Nanodrop.

A total of 500 ng of total cellular RNA or 5 μl of cell culture supernatant isolated vRNA was reverse transcribed (RT) to cDNA. The RT-PCR reaction mix contained 1x RT buffer (Applied Biosystems), 5.5 mM MgCl (Applied Biosystems), 2 mM of dNTP (0.5 mM each) (Applied Biosystems), 2.5 μM random hexamers (Invitrogen), 1 U/μl RNAse inhibitor (Applied Biosystems) and 1.25 U/μl RT enzyme (Thermo Fisher Scientific). A standard cDNA synthesis program was used for the reaction.

To determine relative cellular vRNA expression and mRNA expression levels of target genes, qPCR on cDNA was carried out using TaqMan Gene Expression Master Mix (Applied Biosystems) with primer and probe mixes for SARS-CoV-2 E gene (28), IFN-α1 (Hs00256882_s1), IFN-β1 (Hs01077958_s1), IFN-λ1 (Hs00601677_g1), IFN-λ2 (Hs00820125_g1), CXCL10 (Hs00171042_m1), CCL5 (Hs00174575_m1), IL-6 (Hs00174131_m1), IL-8 (Hs00174097_m1),TNF-α (Hs00174128_m1) and TGF-β (Hs99999918_m1) (all from Applied Biosystems). Target mRNA expression levels were normalized against human 18S (Ribosomal RNA Control Reagents VIC™ Probe, Applied Biosystems) and the 2-ΔΔCt method was used to calculate mRNA expression levels as a relative increase in mRNA compared to the uninfected mock samples.

For the quantification of SARS-CoV-2 vRNA in cell culture supernatant samples, 5 µl of cDNA was amplified with PCR using the TaqMan Gene Expression Master Mix (Applied Biosystems) and the above SARS-CoV-2 E gene primer and probe mix. To quantify viral RNA, the assay included a standard curve of known concentrations (10^1^ - 10^7^) of hCoV-Fin-E-pEBB-HA-N plasmid. The plasmid contains a GeneArt (Thermo Fisher Scientific) synthesized SARS-CoV-2 E gene, based on the hCoV-19/Finland/1/2020 sequence (GenBank MT020781), cloned into pEBB-N-HA mammalian expression vector. The Ct-value of the sample was compared to the ct-value of the known concentrations of the standard curve and the absolute quantity of SARS-CoV-2 in the PCR mix was extrapolated. From this the vRNA quantity in the initial sample was calculated.

### Antibodies

In-house produced polyclonal antibodies were used for immunoblotting and they included rabbit antibodies against SARS-CoV N (cross-reactive against SARS-CoV-2 N) and SARS-CoV-2 S1 proteins (25), MxA protein (29) and IRF3 protein (30). Antibodies from Cell Signaling Technology included Phospho-IRF3 (Ser396) (4D4G) Rabbit mAb (P-IRF3; 4947), p38 MAPK Antibody (9212), Phospho-p38 MAPK (Thr180/Tyr182) Antibody (P-p38; 9211), IκB-α (44D4), Stat2 (D9J7L) Rabbit mAb (72604), Phospho-Stat2 (Tyr690) (D3P2P) Rabbit mAb (88410) and Glyceraldehyde-3-phosphate dehydrogenase (GAPDH; 2118). IFITM3 antibody was from Abgent (#AP1153a). The secondary antibody used was Horseradish peroxidase (HRP)-conjugated goat anti-rabbit antibody (Dako).

### Immunoblotting

Cells were lysed in Passive Lysis Buffer (Promega) supplemented with 1 mM Na_3_VO_4_. Total protein concentrations were determined using the Bio-Rad protein assay. SDS-PAGE was used to separate equal amounts of protein (10 µg -20 µg) which was followed by transfer of the proteins onto Hybond-P polyvinylidene difluoride (PVDF) membranes (Amersham Biosciences). For in-house antibodies, membranes were blocked for 30 min at room temperature (RT) in blocking buffer (5% fat-free milk in PBS with 0.05% Tween-20). Primary and secondary antibodies were diluted in blocking buffer and membranes were incubated for 1h at RT. All washes were carried out for 3 x 10 min in PBS with 0.05% Tween-20. For commercial antibodies blocking and staining were performed according to manufacturer’s instructions. Protein bands were visualised using Pierce™ ECL Western Blotting Substrate (Thermo Fisher) and BIOMAX XAR films (CareStream). Quantification of immunoblots was carried out using ImageJ software (31).

### Sequencing

Sequencing was carried out with Illumina Novaseq 6000 and the data was collated using the HAVoC pipeline (32). The pangolin tool (33) was used to assign the SARS-CoV-2 lineages. Sequence alignments were done with Clustal Omega (34) and sequence analyses were done with BioEdit 7.0.5.3 (35).

### Phylogenetic analysis

For phylogenetic analysis, reference sequences for the variants in this study were obtained from GISAID (https://www.gisaid.org). The reference sequences and sequences of variants in this study were aligned using Clustal Omega (36). A phylogenetic tree was constructed using Molecular Evolutionary Genetics Analysis software (MEGA (37), Version 10.0.5). The best fit model was first determined and the phylogenetic tree was constructed using the Maximum Likelihood method and Tamura-Nei model (38).

### Statistics

GraphPad Prism 9.0 (GraphPad Software, San Diego, CA, USA.) was used for statistical analyses. One-way ANOVA with Tukey’s multiple comparisons test was used.

## Results

### Description of the mutations in pre-Omicron SARS-CoV-2 variants used in this study

**Figure 1:**
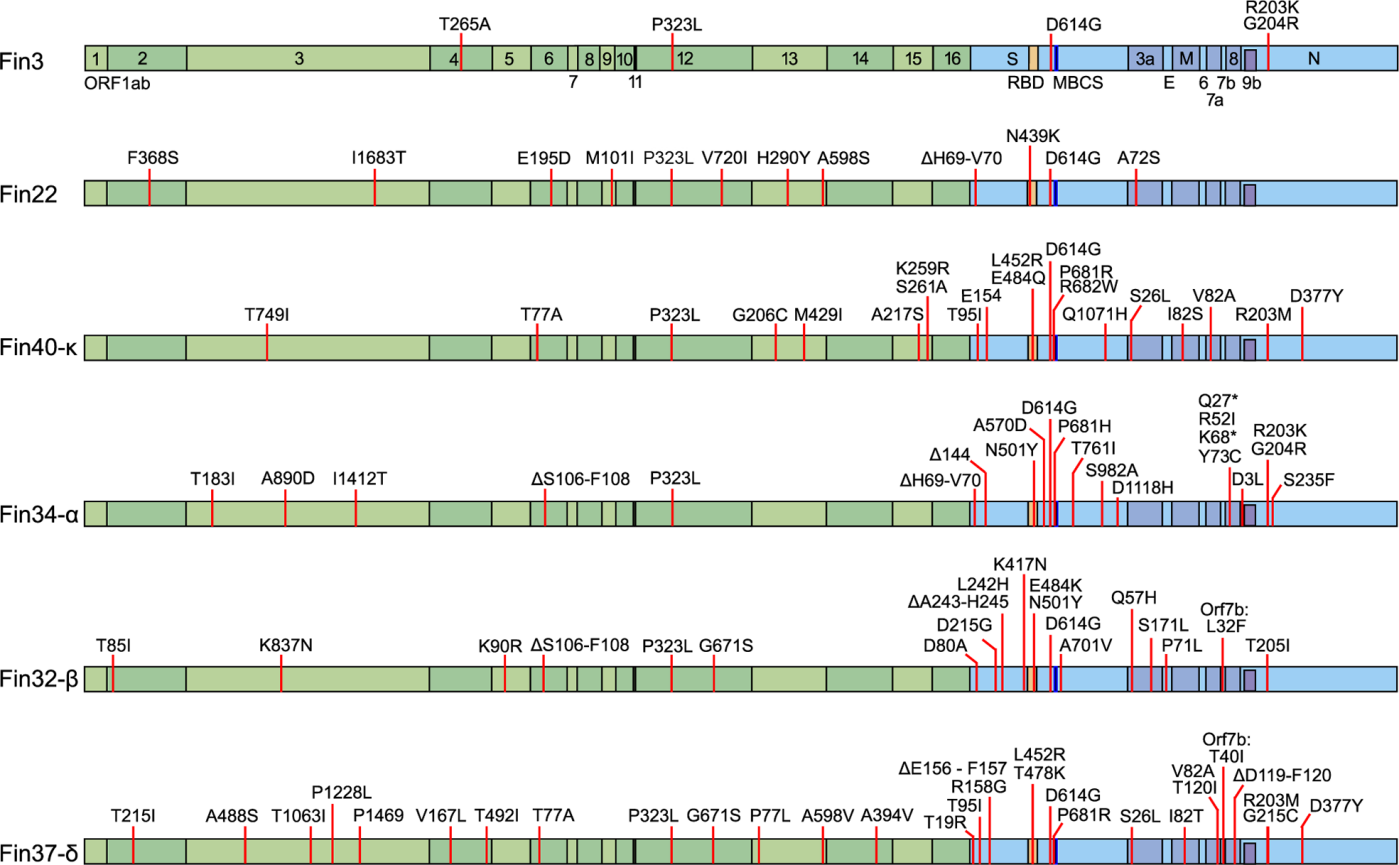
Mutations in the six pre-Omicron SARS-CoV-2 variants used to infect Calu-3 cells. The mutations are mapped against the hCoV-19/Wuhan/WIV04/2019 reference genome (EPI_ISL_402124). In green are the non-structural proteins located in Orf1ab and in blue are the structural proteins S, E, M, N, and accessory proteins. Receptor-binding domain (RBD, S protein amino acid residues 437-507, orange), multi-basic cleavage site (MBCS, S protein amino acid residues 681-685, (P-R-R-A-R), dark blue) are also marked.

All the SARS-CoV-2 variants in this study were isolated from patient samples. The original sample and the virus stocks were whole genome sequenced to confirm the identity of the variants and compare the mutations to the original Wuhan SARS-CoV-2 reference genome (**Figure 1**). Fin3 (Pangolin lineage B.1.1.29) is an early pandemic variant from March 2020 with only five mutations. The P323L and D614G mutations in Nsp12 (RdRp) and S protein, respectively, are present in the other variants as well. In addition to these, the Fin3 N protein contains R203K and G204R mutations, which are also seen in Alpha variants such as in Fin34-α. Fin22 (B.1.258) is from November 2020 and has accumulated a total of 12 mutations. Fin22 is the only variant in this study that does not harbor mutations in the N protein gene. Fin34-α (B.1.1.7) and Fin32-β (B.1.351) were the first VOCs and these viruses were isolated from patient samples collected in January 2021. Fin32-β has 23 mutations while Fin34-α contains a total of 25 mutations. Both have nine amino acid substitutions in the S protein. In the RBD, both Fin32-β and Fin34-α contain the N501Y mutation, while Fin32-β has an additional E484K mutation. Fin34-α harbors a P681H mutation in the MBCS. In the N protein, there is a T205I mutation in Fin32-β at a similar position to the R203K-G204R seen in Fin3 and Fin34-α. The N protein of Fin34-α also contains two additional mutations, D3L and S235F. Fin37-δ (B.1.617.2) and Fin40-κ (B.1.617.1) were isolated from patient samples collected in May 2021 and March 2021, respectively. Although the two strains are genetically close to each other, Fin40-κ has only 21 mutations, while Fin37-δ has 30. Both viruses contain eight mutations in the S protein of which some are the same. Both variants contain a L452R mutation in the RBD and a P681R mutation in the MBCS. There is also a R682W mutation in Fin40-κ, which can occur following viral passage in Vero-E6 cells. In the N protein, the first mutation in Fin37-δ is G215C and both Fin37-δ and Fin40-κ variants contain R203M and D377Y substitutions. Phylogenetic analysis showed Fin22 to cluster near the original Wuhan sequence, while Fin3 clustered nearer the Alpha variant (**Supplementary Figure 1**). The VOCs clustered with their reference counterparts as expected.

### SARS-CoV-2 variants show different replication profiles in human lung epithelial Calu-3 cells

**Figure 2:**
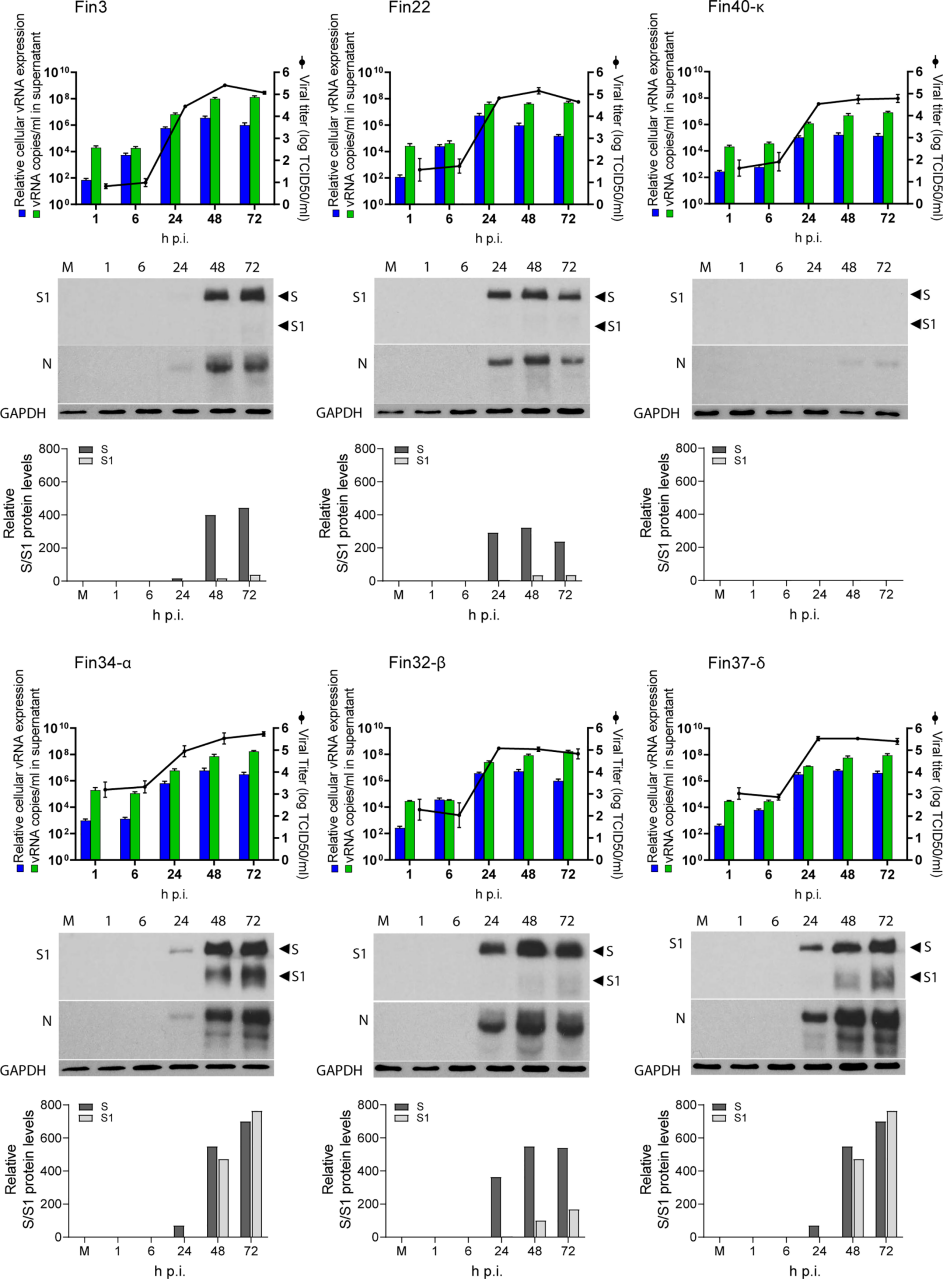
Replication of six SARS-CoV-2 variants in Calu-3 cells. Cells were infected with each variant at a MOI of 1 TCID_50_/cell. Cell culture supernatant and total cellular RNA and protein samples were collected at 1, 6, 24, 48 and 72 hours post-infection (h p.i.). The figure shows the replication profiles for each variant. The relative cellular vRNA expression levels and the quantified vRNA copies/ml in cell culture supernatant (as determined by RT-qPCR) is shown in the graphs on the left Y-axis. The production of infectious virions (as determined by an end point dilution assay and shown as log TCID_50_/ml) is shown on the right Y-axis. The results are the mean values ± SEM of three independent experiments. Cellular protein samples were analyzed by immunoblotting with anti-SARS-CoV-2 S1 (S1) specific and cross-reactive anti-SARS-CoV N protein (N) specific antibodies to show the replication kinetics of the viruses at a protein level. Immunoblotting was carried out once for S protein and three times for N protein. Representative immunoblots are shown. The anti-SARS-CoV-2 S1 antibody recognizes both the full-length S protein (S) and the cleaved S1 fragment. GAPDH was used as a loading control. The blots were exposed with an equal exposure time. S protein amounts quantified using ImageJ are shown below the immunoblots.

Calu-3 cells were infected with different variants at MOI 1 and cellular vRNA levels were determined by RT-qPCR for up to 72 hours (**Figure 2**). Overall, Fin3 and Fin34-α replication patterns were similar as cellular vRNA levels increased with a slow kinetics and maximal vRNA levels were reached within 48 h p.i. Fin22, Fin32-β and Fin37-δ, showed faster replication kinetics and submaximal/maximal vRNA levels were seen already at 24 h p.i., after which Fin32-β and Fin37-δ replication reached a plateau while Fin22 cellular vRNA levels decreased. Fin40-κ replication was consistently an order of a magnitude lower compared to the other variants.

Viral RNA copies in the cell culture supernatant were also quantified (**Figure 2**). Cell culture supernatant vRNA patterns generally followed those of cellular vRNA levels. Importantly, the supernatant vRNA quantities were almost identical at 1h indicating roughly equal amounts of input stock viruses. For Fin34-α, the mean of three independent experiments was slightly higher than for the rest of the variants, nevertheless, the difference was not statistically significant when analyzed with the one-way ANOVA with Tukey’s multiple comparisons test, which confirmed the input virus amounts were correctly normalized.

The endpoint dilution assay was done in VeroE6-TMPRSS2-H10 cells to assess the amount of infectious virus produced from Calu-3 cells at different time points after infection (**Figure 2**). The patterns of infectious virus release mainly correlated with the cellular and supernatant vRNA levels, and they were relatively similar in all variants. Although Fin34-α infected cells measured lower levels of cellular and supernatant vRNA at 24 h p.i., the viral titers were comparable to those of Fin22, Fin32-β and Fin37-δ. Mirroring the vRNA levels, the infectivity of Fin40-κ remained at a lower level.

An additional observation was made while carrying out endpoint dilution assays. CPE was easily identified at three days post infection for all variants except Fin37-δ, which consistently replicated with a slower kinetics in VeroE6-TMPRSS2-H10 cells. Fin37-δ required a longer incubation (six days) for reliable determination of its infectivity titer. The amount of CPE of the other variants did not significantly change after three days of incubation. Due to this the TCID_50_/mL results for the infection assay supernatant samples were read at six days p.i. for all variants.

Western blot analyses (**Figure 2**) showed S and N protein expression in all variants apart from Fin40-κ, which agrees with the lower virus amounts in cells infected with this variant. The relatively slower replication kinetic of Fin3 and Fin34-α variants was also seen at protein level as lower expression of S and N proteins was detected at 24 h p.i. Likewise, Fin22, Fin32-β and Fin37-δ viral protein expression was clearly visible already at 24 h p.i although quantification of the S protein immunoblot showed a lower level of Fin37-δ S protein at this time point (**Figure 2**). Interestingly, S protein cleavage was very efficient for both Fin34-α and Fin37-δ compared to the other variants (**Figure 2**).

### SARS-CoV-2 variants elicit a low level of IRF3, p38 and NF-κB pathway activation but induce strong STAT2 activation

**Figure 3:**
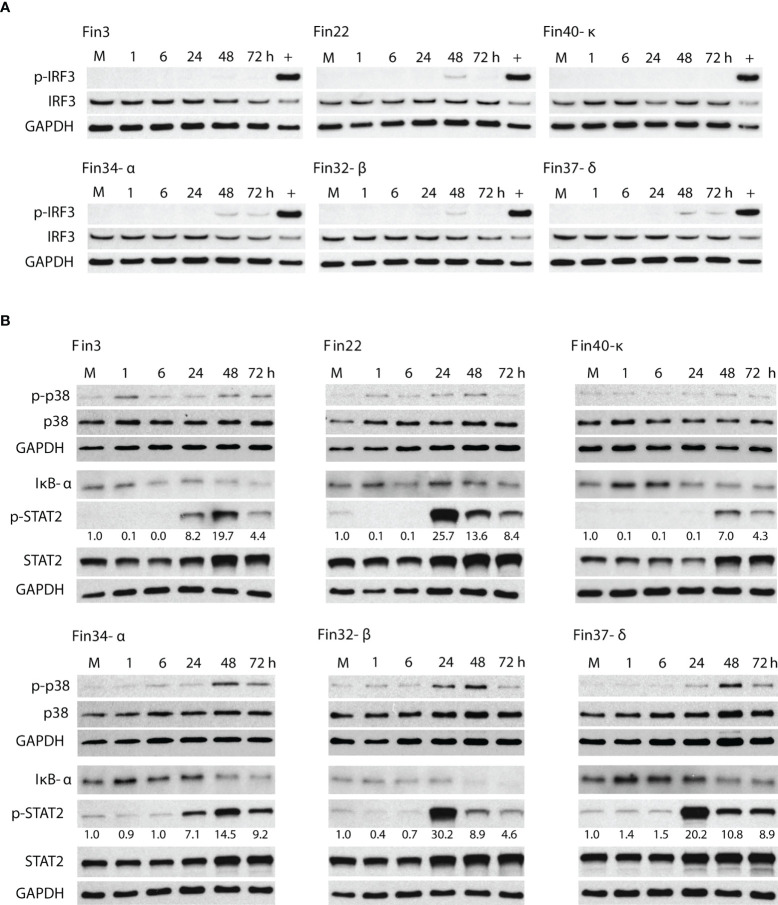
Activation of signaling molecules involved in the induction of interferon, cytokine and ISG gene expression. Total cellular protein samples were collected at various time points following infection of Calu-3 cells with different SARS-CoV-2 variants at a MOI of 1 TCID_50_/cell. Representative immunoblots out of 3 repeated experiments are shown. **(A)** Immunoblots were probed with antibodies against phosphorylated interferon regulatory transcription factor 3 (p-IRF3) and total IRF3. Cellular protein samples collected at 8 h after Sendai virus infection in Calu-3 cells was used as a positive control (+). GAPDH was used as a loading control for p-IRF3. The immunoblot was carried out twice. **(B)** Immunoblots stained with antibodies against phosphorylated p38 (p-p38) and p38 (carried out once), nuclear factor of kappa light polypeptide gene enhancer in B-cells inhibitor alpha (IκB-α) and phosphorylated signal transducer (carried out twice) and activator of transcription 2 (p-STAT2) and STAT2 (carried out twice). p-STAT2 levels were quantified by ImageJ and the fold over mock values are seen below the p-STAT2 immunoblot. GAPDH was used as a loading control.

Activation of various innate immune signaling pathways was assessed by immunoblotting. A very low level of IRF3 phosphorylation was elicited by Fin22 and Fin32-β at 48 h p.i. and by Fin34-α and Fin37-δ at 48 and 72 h p.i. (**Figure 3A**). Phosphorylated IRF3 was hardly detectable in Fin3 infected cells, while no p-IRF3 was seen in Fin40-κ infected cells. The phosphorylation of p38 was also weak and mainly seen at 48 h p.i. in cells infected by the VOCs Fin34-α, Fin32-β and Fin37-δ (**Figure 3B**). A decrease in the level of NF-κB inhibitor IκB-α was observed in Fin34-α, Fin32-β, Fin37-δ infected cells at 48 h p.i. and in the Fin40-κ infection already at 24 h p.i. A decrease in IκB-α was also seen in cells infected with Fin3 and Fin22 at 72 h p.i. (**Figure 3B**). Overall, these results show relatively weak activation of signaling pathways involved in interferon and cytokine gene expression.

On the other hand, a robust activation of the interferon stimulated STAT2 pathway was seen (**Figure 3B**). High levels of p-STAT2 were observed in Fin22, Fin32-β, Fin37-δ infected cells at 24 h p.i. after which the levels decreased. Cells infected with Fin3 and Fin34-α showed a slower phosphorylation kinetics with a peak in p-STAT2 level at 48 h p.i. Only weak STAT2 activation was detected for Fin40-κ. The phosphorylation of STAT2 seemed to occur simultaneously with virus replication (**Figure 2**) as the highest p-STAT2 levels were seen when the cellular vRNA levels reached high levels.

### The expression of interferons and cytokine genes correlates with peak cellular viral RNA levels

**Figure 4:**
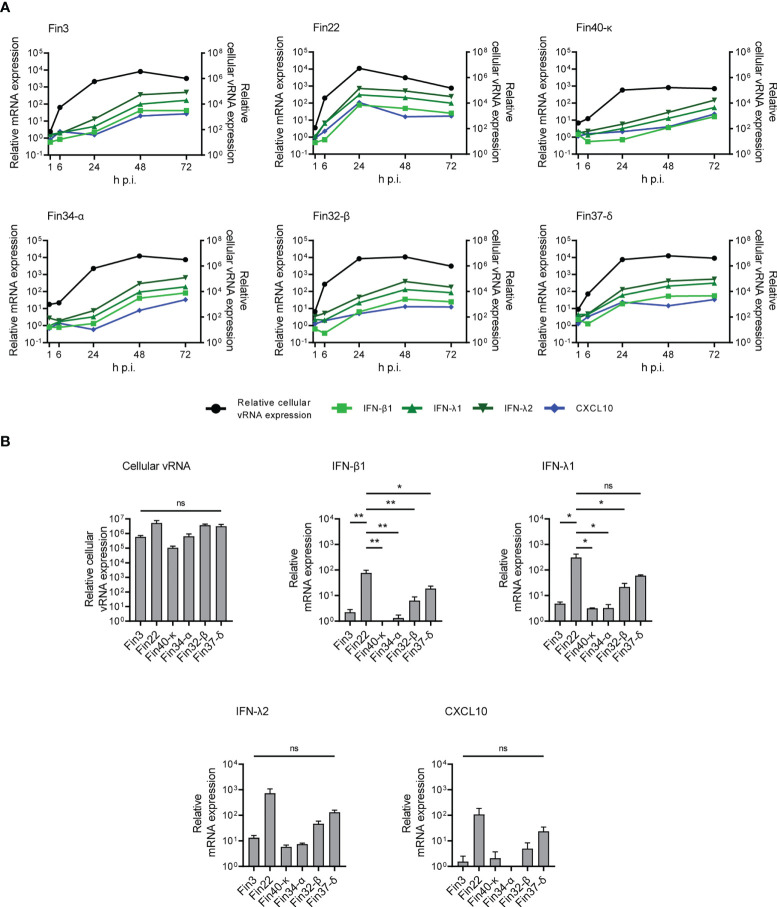
Interferon and CXCL10 mRNA expression levels in Calu-3 cells infected with SARS-CoV-2 variants. Cells were infected with each variant at a MOI of 1 TCID_50_/cell and total cellular RNA samples were collected at different time points during infection. **(A)** The kinetics of vRNA and host cell IFN-β1, IFN-λ1, IFN-λ2 and CXCL10 mRNA expression profiles were determined by RT-qPCR. Relative cytokine mRNA expression profiles in different SARS-CoV-2 variant infected cells are shown on the left Y axes and the vRNA expression levels on the right Y axes. The means of three independent experiments are shown. **(B)** Comparative analysis of relative cellular vRNA and IFN-β1, IFN-λ1, IFN-λ2 and CXCL10 mRNA expression at 24 h p.i. The results are the mean values ± SEM of three independent experiments. One-way ANOVA with Tukey’s multiple comparisons test was used for the statistics. *P* < 0.05 (*), *P* < 0.01 (**), not significant (ns).

The expression of interferon and cytokine genes was assessed at mRNA expression level with RT-qPCR. IFN-β1, IFN-λ1, IFN-λ2 and CXCL10 mRNA expression was detected (**Figure 4A**; **Supplementary Figure 2** and **Supplementary Table 1**), while no increased expression of IFN-α, TNF-α, IL-6, IL-8, TFG-β and CCL5 mRNAs was observed (data not shown).

The expression levels of IFN-β1, IFN-λ1, IFN-λ2 and CXCL10 mRNAs were dependent on cellular vRNA levels although some differences in the cytokine mRNA expression patterns were seen in cells infected by different variants (**Figure 4A**). In Fin22 infected cells, IFN and CXCL10 mRNA expression levels peaked with cellular vRNA levels at 24 h p.i. In contrast, in Fin3, Fin34-α, Fin32-β and Fin37-δ infected cells IFN and CXCL10 mRNA levels did not peak until 48 h p.i. when cellular vRNA levels had reached a plateau. A weaker and slower IFN and CXCL10 mRNA expression pattern was seen in Fin40-κ infected cells, which was consistent with the lower viral replication levels.

The variation in viral replication levels at 24 h p.i. was not significant (**Figure 4B**), however, the level of IFN-β1 expression was significantly higher in cells infected with Fin22 compared to the other variants. IFN-λ1 mRNA levels were also significantly higher for Fin22 infected cells compared to those of Fin3, Fin34-α, Fin32-β and Fin40-κ infected cells. Although IFN-λ2 and CXCL10 mRNA expression patterns were similar, the differences were not statistically significant.

### Induction of ISGs by SARS-CoV-2 infection is sensitive to IFNs

**Figure 5:**
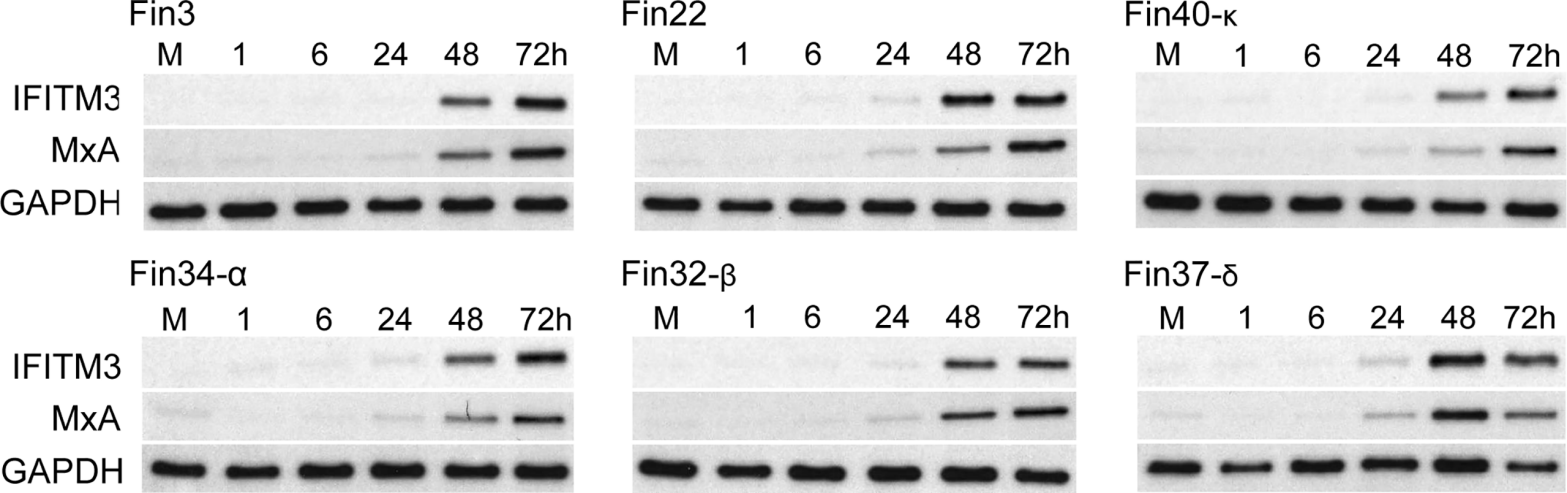
Kinetics of expression of antiviral ISGs in Calu-3 cells infected with different SARS-CoV-2 variants. Six different virus variants were used at MOI of 1 TCID_50_/cell to infect Calu-3 cells and during the 3-day infection cellular protein samples were collected. Representative immunoblots were probed with antibodies against interferon induced transmembrane protein 3 (IFITM3) (carried out once) and human Myxovirus resistance protein A (MxA) (carried out twice). GAPDH was used as a loading control.

Secreted type I and type III IFNs induce the expression of ISGs such as IFITM3 and MxA proteins that mediate the antiviral actions against multiple viruses. The expression of IFITM3 and MxA proteins during SARS-CoV-2 infection in Calu-3 cells was analyzed by immunoblotting. All variants stimulated almost similar amounts of IFITM3 and MxA production in infected Calu-3 cells (**Figure 5**). A weak increase in ISGs was seen already at 24 h p.i. but the highest levels were observed at 48 and 72 h p.i. ISG expression levels were relatively similar regardless of the levels of cellular vRNAs, different kinetics and strength in the activation of cellular signaling pathways and interferon mRNA expression levels. In Fin40-κ infected cells almost similar amounts of IFITM3 and MxA protein expression was seen compared to the other variant infected cells even though the replication, interferon mRNA expression and STAT2 phosphorylation by Fin40-κ occurred at a lower level. On the other hand, Fin22, Fin32-β and Fin37-δ, which induced high levels of p-STAT2 activation, did not induce much higher levels of ISGs than Fin40-κ. Thus, the induction of IFITM3 and MxA is highly sensitive to even small amounts of IFNs produced.

### Omicron sublineage BA.2 does not replicate as well as BA.1 or the recombinant sublineage XJ in Calu-3 cells

**Figure 6:**
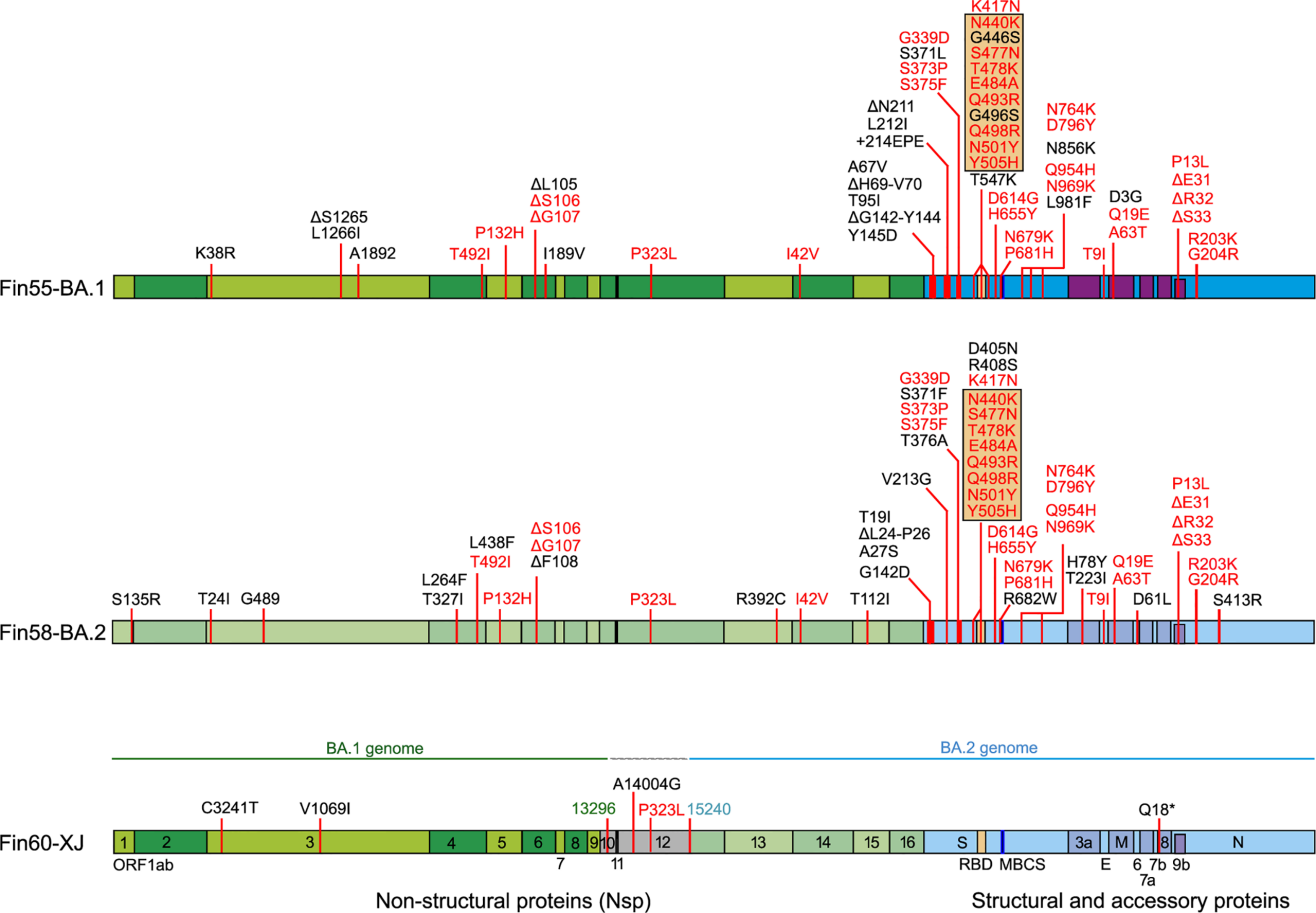
Mutations in three Omicron sublineages, Fin55-BA.1, Fin58-BA.2 and recombinant Fin60-XJ. The hCoV-19/Wuhan/WIV04/2019 reference genome (EPI_ISL_402124) was used to map the mutations. In black are unique mutations and in red are mutations that are found in all the above Omicron sublineages. Mutations in the orange box are found in the RBD. Fin60-XJ is a recombinant of BA.1 and BA.2 with a cut off between nucleotides 13 296 (green) in Nsp10 and 15 240 (blue) in Nsp12. Receptor-binding domain (RBD, S protein amino acid residues 437-507, orange), multi-basic cleavage site (MBCS, S protein amino acid residues 681-685, (P-R-R-A-R), dark blue).

Replication and interferon induction of three Omicron sublineages Fin55-BA.1, Fin58-BA.2 and recombinant Fin60-XJ was also studied. Fin55-BA.1 was isolated from a patient sample collected in December 2021 and whole genome sequencing confirmed all 62 defining mutations of the BA.1 sublineage (**Figure 6**). Fin58-BA.2 and Fin60-XJ patient samples were from January 2022. Fin58-BA.2 contained the 62 defining mutations in addition to a H78Y mutation in ORF3a, which has been seen in some BA.2 sublineages (39). Fin58-BA.2 also contained the R682W mutation in the S protein. Fin60-XJ is a recombinant sublineage in which the 5’ end is from BA.1 and a cut off between nucleotides 13,296 and 15,240 in Nsp10 and Nsp12, respectively, switches the genome to BA.2 (40). Fin60-XJ did not have the R682W mutation seen in Fin58-BA.2. All the three Omicron sublineages harbored the P681H mutation in the S protein MBCS and the R203K/G204R mutations in N protein, which are seen in the Alpha variant as well. A phylogenetic analysis showed that Fin55-BA.1 and Fin58-BA.2 clustered with their reference counterparts and Fin60-XJ clustered between these two (**Supplementary Figure 1**).

**Figure 7:**
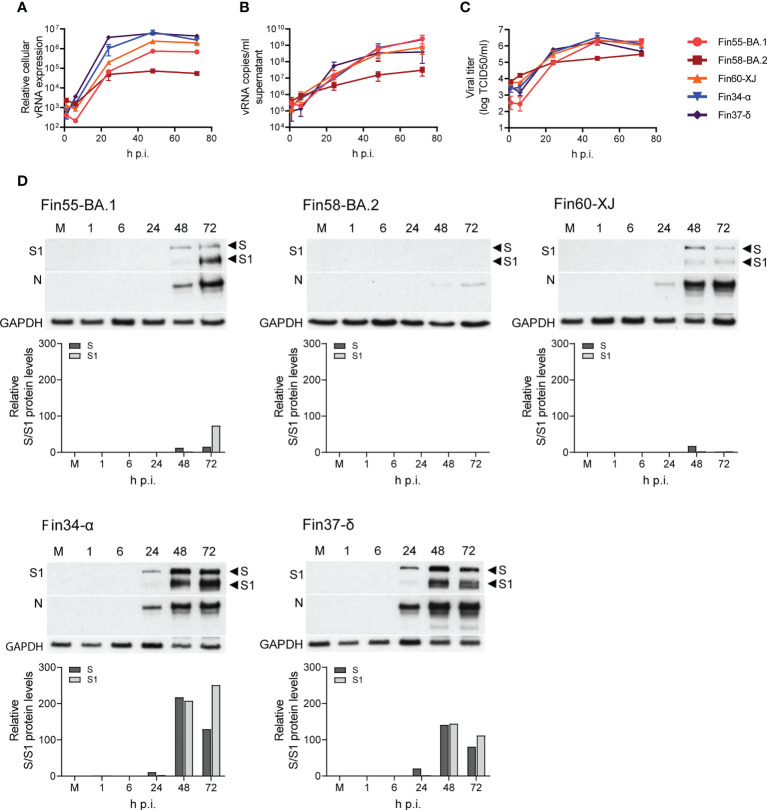
Replication of Omicron sublineages BA.1, BA.2 and XJ compared to Alpha and Delta. Calu-3 cells were challenged with three Omicron sublineages and Alpha and Delta variants at multiplicity of 1 for three days and different samples were collected for analysis of the viral molecules. **(A)** Relative cellular vRNA expression levels determined by RT-qPCR of Fin55-BA.1, Fin58-BA.2 and Fin60-XJ compared to Alpha (Fin34-α) and Delta (Fin37-δ). **(B)** Cell culture supernatant vRNA copies/ml were quantified by RT-qPCR. **(C)** An end point dilution assay was carried out to determine the production of infectious virions. Results shown as log TCID_50_/ml. **(D)** Immunoblot analysis of SARS-CoV-2 S protein expression by anti-SARS-CoV-2 S1 fragment antibody (S1) and N protein expression using a cross-reactive anti-SARS-CoV-N protein antibody (N) were carried out once. Full length S protein (S) and the cleaved S1 fragment (S1) are marked with arrows. S protein amounts quantified using ImageJ are shown in graphs below the immunoblots. GAPDH was used as a loading control. The qPCR and end point dilution assay results are the mean values ± SEM of three independent experiments.

Calu-3 cells were infected with Fin55-BA.1, Fin58-BA.2, Fin60-XJ, Fin34-α and Fin37-δ at a MOI of 1 and replication was observed for 72 hours. The intracellular vRNA levels of the Omicron sublineages, as determined by RT-qPCR, were lower than those for the Alpha and Delta variants (**Figure 7A**). Fin55-BA.1, Fin58-BA.2 and Fin60-XJ replication was similar up to 24 h p.i. after which Fin58-BA.2 reached a plateau. Interestingly, the recombinant variant Fin60-XJ had a replication profile more like Fin55-BA.1 as it replicated better than Fin58-BA.2 at later time points. The vRNA copies/ml in the cell culture supernatant (**Figure 7B**), however, were lower only for Fin58-BA.2. Consistent with this, an end point dilution assay carried out in VeroE6-TMPRSS2-H10 cells showed that Fin58-BA.2 also produced less infectious virus (**Figure 7C**).

S and N protein expression, as determined by immunoblotting, correlated with replication levels as less protein was detected with the Omicron sublineages (**Figure 7D**). Quantification of the S protein immunoblots (**Figure 7D**) showed that as with Fin34-α and Fin37-δ, efficient cleavage of Fin55-BA.1 and Fin60-XJ S protein was observed especially at 72 h p.i. S protein levels of Fin58-BA.2 were too low to detect the cleavage state.

### Omicron sublineages show a similar slow interferon induction type as other VOCs

**Figure 8:**
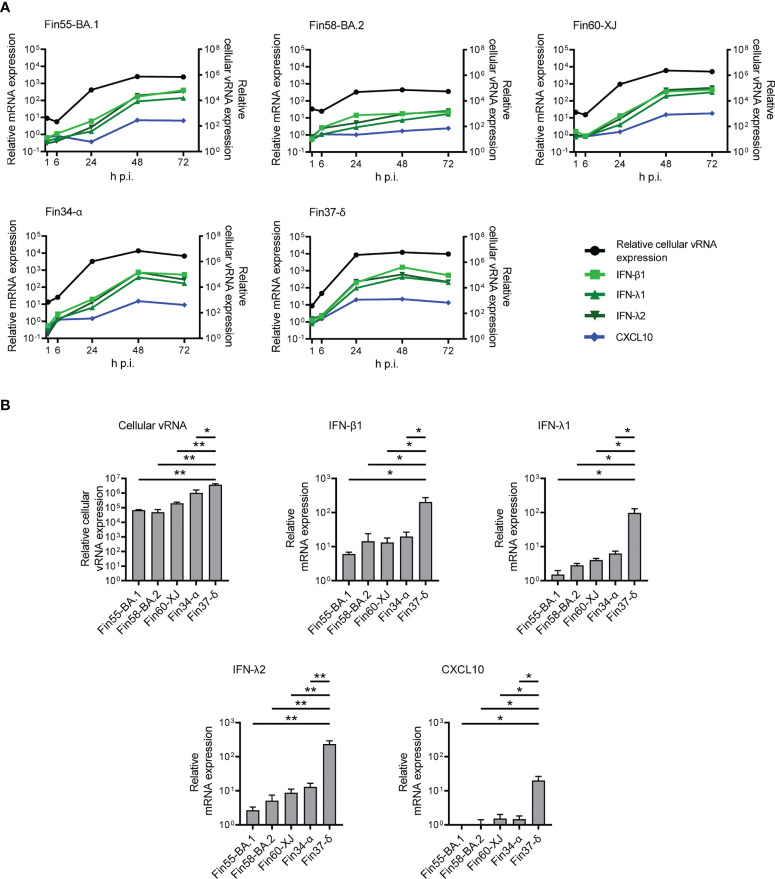
Interferon and CXCL10 mRNA expression levels in Calu-3 cells with Omicron infection. The Omicron sublineages BA.1, BA.2 and recombinant XJ as well as the Alpha and Delta variants were used for infecting Calu-3 cells at a MOI of 1 TCID_50_/cell, and total RNA samples were collected at different time points for RT-qPCR analysis. **(A)** IFN-β1, IFN-λ1, IFN-λ2 and CXCL10 mRNA expression profiles determined by RT-qPCR (left Y axis) compared to the relative cellular vRNA expression profile (right Y axis) for cells infected with three Omicron variants (Fin55-BA.1, Fin58-BA.2 and Fin60-XJ), and with Alpha (Fin34-α) and Delta (Fin37-δ). The means of three independent experiments are shown. **(B)** Comparison of relative cellular vRNA and IFN-β1, IFN-λ1, IFN-λ2 and CXCL10 mRNA expression at 24 h p.i. The results are the mean values ± SEM of three independent experiments. One-way ANOVA with Tukey’s multiple comparisons test was used for the statistics. *P* < 0.05 (*), *P* < 0.01 (**).

The mRNA expression levels of interferons and CXCL10 were determined with RT-qPCR. In concordance with lower vRNA expression, the mRNA expression levels of IFN-β1, IFN-λ1, IFN-λ2 and CXCL10 were lower for cells infected with Fin58-BA.2 than cells infected with Fin55-BA.1, Fin60-XJ, Fin34-α and Fin37- δ (**Figure 8A**; **Supplementary Figure 3** and **Supplementary Table 2**). The greatest difference in interferon expression levels was observed at 24 h p.i., as IFN-β1, IFN-λ1, IFN-λ2 and CXCL10 mRNA expression levels were significantly lower in cells infected with Omicron subvariants and Fin34-α compared to Fin37-δ infected cells (**Figure 8B**). However, cellular vRNA levels were also significantly lower for these variants compared to Fin37-δ.

## Discussion

The aim of this study was to compare SARS-CoV-2 variants isolated from different epidemic peaks of the pandemic, to see whether there are differences in the replication and innate immune responses in genetically distinct SARS-CoV-2 viruses and whether we could relate the potential differences to some specific mutations in the genome. SARS-CoV-2 has been reported to mutate at a rate of 4 x 10^-4^ - 2 x 10^-2^ mutations per nucleotide per year (41). The majority of these mutations are neutral or even deleterious, nonetheless, some mutations do arise that increase viral fitness and immune evasion (42). Genotype to phenotype studies of viruses may provide us understanding of which changes in the genome are important for viral transmissibility and virulence. SARS-CoV-2 offers a unique opportunity to study this, since already 10 million SARS-CoV-2 genome sequences have been uploaded to the Global Initiative on Sharing All Influenza Data (GISAID) website (43) as of spring 2022.

Here we compared the ability of nine genetically different SARS-CoV-2 variants to replicate and induce innate immunity in a human lung epithelial cell model. Overall, the pre-Omicron variants replicated in a comparable fashion, however, Fin34-α replication took place at somewhat slower kinetics than Fin32-β and Fin37-δ, and the Kappa variant, Fin40-κ, replicated at ten-fold lower levels compared to the other variants. A slower replication pattern of the Alpha variant compared to Delta in Calu-3 cells was also shown by Mlcochova and co-workers (44). Elsewhere, the replication of Alpha, Beta and early pandemic variants in Calu-3 have shown almost identical growth characteristics (24, 45, 46). The Omicron BA.1 variant has been shown to replicate less efficiently in lower respiratory tract cells, including Calu-3 (46–49). We also observed that replication levels of BA.1 and a recombinant variant XJ in Calu-3 were an order of magnitude lower compared to Alpha and Delta, while replication of a BA.2 variant was two log lower at later time points. Hence, we can conclude that compared to Omicron, the earlier VOCs show only some minor variation in their replication kinetics in Calu-3 cells.

Studies on the effects of single mutations on viral replication have often been conducted computationally or with recombinant proteins, pseudoviruses and WT viruses with genetically engineered mutations. At the start of the pandemic, epidemiologic observations, studies on pseudoviruses and genetically engineered SARS-CoV-2 mutants with single mutations showed that the S protein D614G and RdRp P323L mutations rendered the original Wuhan SARS-CoV-2 variant more transmissible (2–5). These results are clearer as the genetic background did not yet contain various other mutations. As the virus evolved, subsequent mutations have been studied in the same way. For example, in the S protein the N501Y mutation in Alpha and Beta variants and E484K in Beta were shown computationally to increase the affinity of the RBD to ACE2 (50–52). Viral transmission and replication was shown to be enhanced in a genetically engineered N501Y mutant with a WT SARS-CoV-2 background (53). In our study with authentic viruses, however, we did not observe a great difference in replication of Fin34-α and Fin32-β, which contain these RBD mutations, compared to Fin3, Fin22 and Fin37-δ, which lack them. In the MBCS, the P681R mutation in Delta and Kappa and the P681H mutation in the Alpha variant have been shown to increase S protein cleavage (54–56) and cell entry compared to the WT virus (44). These studies were done using fluorogenic peptides (54, 56), pseudoviruses (44, 54, 56), and recombinantly generated mutant viruses (55). However, the enhanced cleavage of Alpha, Delta and Kappa S protein did not lead to increased replication of natural viral isolates in Calu-3 cells (55, 56). In concordance with this, we observed similar levels of cleaved S protein in Fin34-α and Fin37-δ which were higher than that seen for Fin32-β and the early pandemic variants, but this did not affect the replication of the viruses in Calu-3 cells. Omicron also has the P681H mutation, and we could clearly see the cleavage of BA.1 and XJ sublineage S proteins in our study. Some studies have shown reduced cleavage of BA.1 S protein, thus hypothesizing that the Omicron S protein is less efficiently cleaved, which could cause the potential shift in the cell entry mechanism (46, 48, 57). However, the reduced cleavage was shown with S protein from pseudovirus (PV) studies (57) and live virus infection of VeroE6-TMPRSS2 cells (46, 48) but not with S protein from live virus infection of Calu-3 cells (47). The effects of the ever-increasing number of S protein mutations are complex and studies to pinpoint roles of certain mutations is challenging as is evident, for example, with the Delta and Kappa variants. Both have many of the same beneficial mutations but the reason for the weaker replication of Fin40-κ or the decreased fitness of the Kappa variant epidemiologically is unclear as studies on the role of different mutations have remained elusive (7, 58, 59). Likewise, while the effects of S protein mutations in Omicron BA.1 and BA.2 on evasion of adaptive immunity are clear (60, 61), their role in the changed replication efficiency in the lower respiratory tract cells is still uncertain. Research on the BA.1 variant have suggested that the poorer replication could be due to the mutations resulting in less efficient use of TMPRSS2 (47, 48, 57). Whether this causes a shift in Omicron to use the endocytic pathway of entry (46, 48, 49, 57), or the S protein MBCS is merely cleaved by a serine protease other than TMPRSS2 and still uses the cell membrane fusion pathway (47) is still to be confirmed. Here we showed that especially the replication of the BA.2 sublineage was hampered compared to BA.1 and the recombinant XJ. The S protein of XJ is the same as in BA.2, hence mutations in BA.2 S protein might not be the cause of the lower replication efficiency of the sublineage.

The significance of mutations outside the S protein are also being increasingly studied. The N protein is an abundant structural protein (62) that has a crucial role in ribonucleocapsid formation and attachment to the viral membrane (20) and it is critical for vRNA replication and transcription (63, 64). The N protein also undergoes liquid-liquid phase separation (LLPS), which facilitates the compartmentalization of viral protein-protein or vRNA-protein interactions (65). Regulation of these functions is *via* phosphorylation of a serine and arginine rich (SR) motif (aa 175-206) in the central intrinsically disordered region (IDR) of the N protein (aa 175-246) (65–69). Within the SR motif is a R185-G204 site that has been shown to mutate more frequently than expected (11). The Delta and Kappa variants harbor a R203M mutation at this site, which was shown to increase virus replication in lung cells (70). In addition, the Alpha variant double mutation R203K/G204R, has been shown to enhance the replication and infectivity of SARS-CoV-2 *in vitro* and *in vivo* (71, 72). Increased phosphorylation of N protein in Alpha (R203K/G204R), Kappa (R203M) and Beta (contains a T205I mutation) variants was suggested to contribute to the replication efficiency of these variants in LLPS compartments (65, 72). The R203K/G204R mutant N protein also showed increased binding to vRNA, and there was differential expression of immune related genes in cells expressing the mutant N protein (73). These studies, however, have again been carried out using genetically modified WT SARS-CoV-2 viruses, pseudoviruses and recombinant proteins. In our study with natural viruses, the effects of the N protein mutations on replication were not clear as the variants' replication patterns did not seem to correlate with the presence of mutations in the protein. The Omicron sublineages, for example, also contain the R203K/G204R mutation and their replication is not enhanced.

In our cell model we observed a weak activation of IRF3, p38 and NF-κB by the pre-Omicron variants. This activation was, however, sufficient to induce interferon gene expression, JAK/STAT activation, and the production of ISGs. The levels of type I and type III interferons and phosphorylation of STAT2 correlated well with cellular vRNA levels but all the variants produced similar levels of ISGs, even the Fin40-κ variant, which showed a weaker ability to replicate compared to other variants. Previously we shown that Fin-25 (D614G/P323L variant) induced better interferon gene expression and MxA protein expression levels compared to a WT Fin-1 variant (25). However, Fin-1 replicated very poorly in Calu-3 cells, so there could be a minimum threshold required for the activation of ISG production. At RNA level, the Alpha variant has been shown to induce lower levels of ISG mRNA expression compared to WT SARS-CoV-2 (24). In a luciferase reporter assay, the N protein was shown to decrease Sendai virus-induced phosphorylation and nuclear translocation of STAT1/STAT2 dimers and thus inhibit the expression of ISGs (74). We, however, did not see an inhibition of STAT2 phosphorylation by any of the studied variants and IFN-induced MxA protein expression was also clearly detectable. This does not formally rule out that N or some other SARS-CoV-2 protein could at least to some extent inhibit the nuclear translocation of STAT1/STAT2 complexes downregulating ISG mRNA expression. This possibility has to be further considered and analyzed by using expression constructs for individual viral genes. Initial analyses have revealed that certain NSPs and accessory proteins may interfere with IFN signaling (75).

There have been reports of delayed immune activation in SARS-CoV-2 infected cells whereby the interferon and cytokine response seems to peak after the most productive replication stage of the virus (24, 76, 77). In our study we observed a similar type of pattern for cells infected with all the variants, including the Omicrons. The only exception was Fin22, in which cellular vRNA and interferon mRNA levels peaked earlier at 24h p.i. Among other SARS-CoV-2 proteins, the N protein has been shown to inhibit interferon gene expression (78), potentially by interacting with RIG-I (79). Mutations in the SARS-CoV-2 genome may have an effect on innate immune responses. For example, the expression of Orf9b, an alternate reading frame nested in the N protein gene sequence, was increased in the Alpha variant-infected cells possibly due to a D3L mutation and may be involved in delaying IFN gene expression by inhibiting TOMM70 interaction with MAVS (24). In accordance with these observations, we also observed weak activation of IRF3 phosphorylation in the pre-Omicron variant-infected cells. Also, a R203K/G204R mutation of SARS-CoV-2 N protein leads to expression of sgRNA N* in Alpha variant-infected cells but the role of N* in virus replication and regulating immune responses is yet to be elucidated (24, 80). It was of interest that Fin22, which lacks the R203K/G204R mutations in N protein, showed a faster kinetics of IFN gene expression compared to the other variants, especially Fin34-α and Omicrons which all harbor the R203K/G204R mutation. However, regardless of the ability of the Fin22 variant to induce IFN gene expression faster and more efficiently, STAT2 phosphorylation and ISG expression occurred equally well in Fin22, Fin32-β and Fin37-δ virus-infected cells. Thus, the significance of potential variation in different variant-induced interferon response is presently unclear.

In conclusion, this comparative study showed that, in human lung epithelial Calu-3 cells, replication of the pre-Omicron VOCs and two early pandemic SARS-CoV-2 variants was similar while that of Kappa and three different Omicron sublineages was less efficient. Several studies analyzing the effects of the SARS-CoV-2 mutations have been done with pseudoviruses and artificial mutants created in a WT SARS-CoV-2 background. While these studies may provide interesting insights on the role of individual mutations, they do not provide a whole picture of the pathogenic characteristics of different variants. Thus, systematic analyses of virus-host cell interactions of different variant-infected cells are well justified. Our results with nine natural virus variants show that the mutations in SARS-CoV-2 variants have complex effects in combination. Highly beneficial mutations likely compensate for unfavorable ones and further research is required to decipher their roles. We also revealed that the activation of innate antiviral immunity occurs relatively late in the infection by all variants except Fin22, which was able to induce the interferon response faster. Fin22 is a variant without mutations in the N protein, which has many roles in replication and host immune responses, thus it could be an interesting research avenue to follow.

## Data availability statement

The datasets presented in this study can be found in online repositories. The names of the repository/repositories and accession number(s) can be found below:

https://www.ncbi.nlm.nih.gov/genbank/, ON531991

https://www.ncbi.nlm.nih.gov/genbank/, ON532015

https://www.ncbi.nlm.nih.gov/genbank/, ON532063

https://www.ncbi.nlm.nih.gov/genbank/, ON532062

https://www.ncbi.nlm.nih.gov/genbank/, ON532078

https://www.ncbi.nlm.nih.gov/genbank/, ON532082

https://www.ncbi.nlm.nih.gov/genbank/, ON532087

https://www.ncbi.nlm.nih.gov/genbank/, ON532088

https://www.ncbi.nlm.nih.gov/genbank/, ON532089

## Author contributions

LL contributed to the methodology, analysis, investigation, data curation, visualization, writing – original draft, writing – review & editing. MS contributed to the methodology, investigation, writing – review & editing. EV contributed to the methodology. IJ contributed to the conceptualization, investigation, resources, funding acquisition, writing – review & editing. PÖ contributed to the conceptualization, methodology, investigation, resources, supervision, funding acquisition, writing – original draft, writing – review & editing. All authors contributed to the article and approved the submitted version.

## Funding

This research was funded by Academy of Finland, grant number 339511 CrossBar project (PÖ) and the Jane and Aatos Erkko Foundation (IJ). This study was supported by THL coordinated funding for Covid-19 research included in the Finnish Government’s supplementary budget. The funders had no role in the conceptualization, investigation and analysis of the study or decision to publish the work.

## Acknowledgments

We thank Tiina Sihvonen, Johanna Rintamäki, Hanna Valtonen, Miao Jiang, Soile Blomqvist, Erika Lindh, Kirsi Liitsola, Katri Keino, Mikko Määttä, Irmeli Iranto and Emilia Salejärvi. We gratefully acknowledge the authors and their respective laboratories, who analyzed and submitted the sequences to GISAID’s EpiCoV and GenBank Database.

## Conflict of interest

The authors declare that the research was conducted in the absence of any commercial or financial relationships that could be construed as a potential conflict of interest.

## Publisher’s note

All claims expressed in this article are solely those of the authors and do not necessarily represent those of their affiliated organizations, or those of the publisher, the editors and the reviewers. Any product that may be evaluated in this article, or claim that may be made by its manufacturer, is not guaranteed or endorsed by the publisher.
