# Supplementary material for: SARS-CoV-2 variants Alpha, Beta, Delta and Omicron show a slower host cell interferon response compared to an early pandemic variant
Source: Front Immunol. 2022 Sep 30;13:1016108. doi: 10.3389/fimmu.2022.1016108 (PMC9561549; doi:10.3389/fimmu.2022.1016108)
Supplement: Supplementary file 1 [file DataSheet_1.docx]

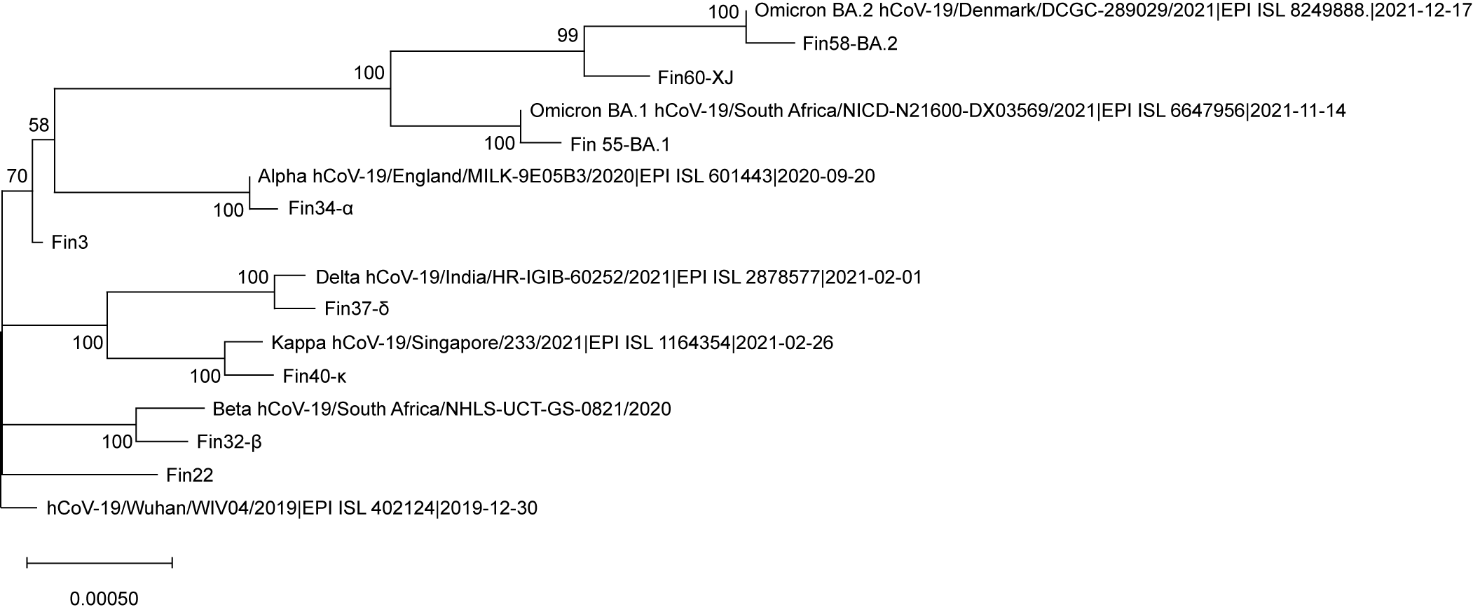


**Supplementary Figure 1. Phylogenetic analysis of all the SARS-CoV-2 variants in this study.** A phylogenetic analysis of the original hCoV-19/Wuhan/WIV04/2019 and all the variants in this study with their representative reference sequences (downloaded from GISAID, https://www.gisaid.org) was carried out using the Molecular Evolutionary Genetics Analysis software (MEGA, Version 10.0.5). The Maximum Likelihood method and Tamura-Nei model were used and 1000 bootstraps was applied.


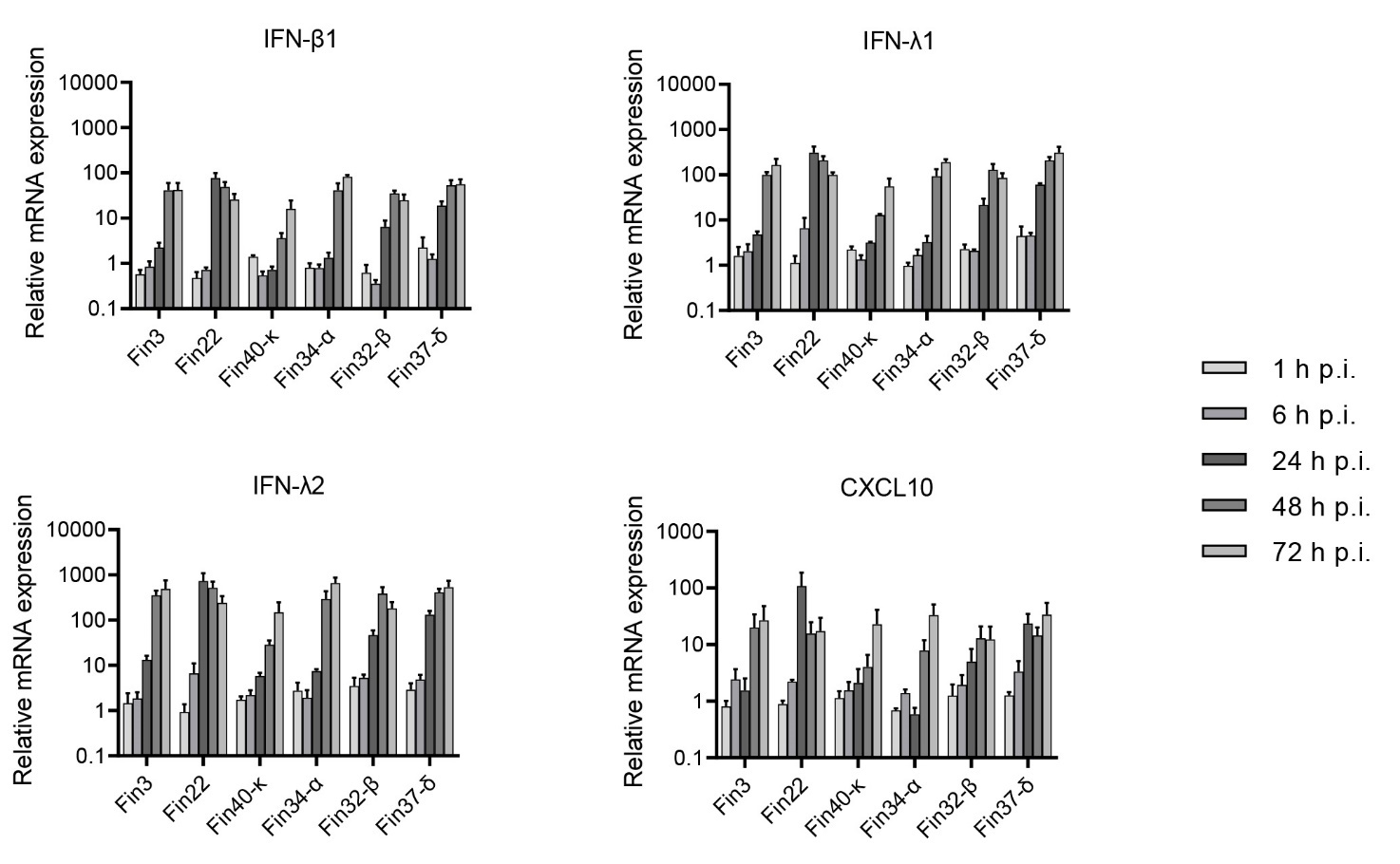


**Supplementary Figure 2.** **Kinetics of IFN-β1, IFN-λ1, IFN-λ2 and CXCL10 mRNA expression in Calu-3 cells infected with SARS-CoV-2 variants.** Calu-3 cells were infected at MOI of 1 TCID_50_/cell with various SARS-CoV-2 variants and the infection was monitored for 72 h. Interferon and CXCL10 levels were determined by RT-qPCR, at various time points following infection. The graphs show the mean values ± SEM of three independent experiments.


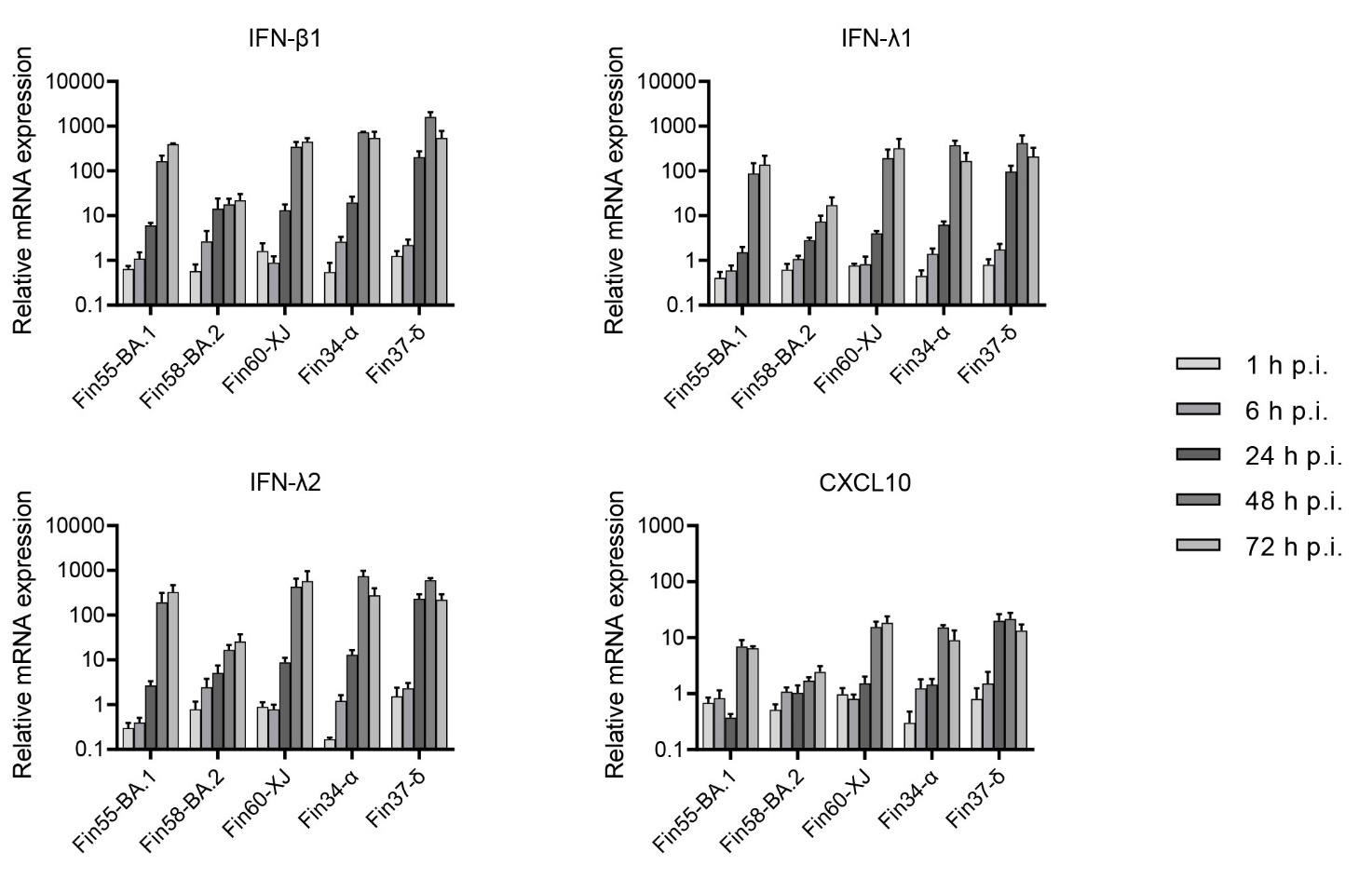


**Supplementary Figure 3. Kinetics of IFN-β1, IFN-λ1, IFN-λ2 and CXCL10 mRNA expression in Calu-3 cells infected with Omicron sublineages and Alpha and Delta variants.** Calu-3 cells were infected at MOI 1 TCID_50_/cell with BA.1, BA.2, recombinant XJ and Alpha and Delta variants. RNA samples were taken at various time points to determine interferon and CXCL10 levels by RT-qPCR. The graphs show the mean values ± SEM of three independent experiments.

|  | **h p.i.** | **Fin3** | **Fin22** | **Fin40-κ** | **Fin34-α** | **Fin32-β** | **Fin37-δ** |
| --- | --- | --- | --- | --- | --- | --- | --- |
| **Relative cellular** | 1 | 7.0E+01 | 1.2E+02 | 2.8E+02 | 1.0E+03 | 2.7E+02 | 4.1E+02 |
| **vRNA expression** | 6 | 5.5E+03 | 2.5E+04 | 6.1E+02 | 1.3E+03 | 3.7E+04 | 6.5E+03 |
|  | 24 | 5.9E+05 | 5.3E+06 | 1.1E+05 | 6.5E+05 | 3.7E+06 | 3.2E+06 |
|  | 48 | 3.6E+06 | 9.8E+05 | 1.6E+05 | 6.0E+06 | 5.2E+06 | 6.3E+06 |
|  | 72 | 1.0E+06 | 1.5E+05 | 1.4E+05 | 3.1E+06 | 9.8E+05 | 4.1E+06 |
| **Relative IFN-β1** | 1 | 0.6 | 0.5 | 1.4 | 0.8 | 0.6 | 2.2 |
| **mRNA expression** | 6 | 0.8 | 0.7 | 0.5 | 0.8 | 0.4 | 1.3 |
|  | 24 | 2.3 | 77.0 | 0.7 | 1.3 | 6.4 | 18.8 |
|  | 48 | 41.3 | 48.8 | 3.6 | 41.2 | 35.2 | 53.3 |
|  | 72 | 42.0 | 25.6 | 16.1 | 82.4 | 24.9 | 56.0 |
| **Relative IFN-λ1** | 1 | 1.6 | 1.1 | 2.2 | 1.0 | 2.3 | 4.4 |
| **mRNA expression** | 6 | 2.0 | 6.6 | 1.3 | 1.7 | 2.1 | 4.5 |
|  | 24 | 4.8 | 308.0 | 3.2 | 3.3 | 21.7 | 60.3 |
|  | 48 | 99.7 | 208.9 | 12.8 | 92.8 | 129.4 | 208.9 |
|  | 72 | 166.1 | 99.8 | 55.6 | 190.3 | 85.2 | 309.1 |
| **Relative IFN-λ2** | 1 | 1.5 | 0.9 | 1.7 | 2.8 | 3.5 | 2.9 |
| **mRNA expression** | 6 | 1.9 | 6.7 | 2.2 | 1.9 | 5.3 | 4.8 |
|  | 24 | 13.2 | 730.4 | 5.8 | 7.5 | 46.5 | 131.0 |
|  | 48 | 352.6 | 516.3 | 28.5 | 293.1 | 385.0 | 412.9 |
|  | 72 | 490.4 | 241.6 | 148.5 | 659.5 | 181.8 | 532.7 |
| **Relative CXCL10** | 1 | 0.8 | 0.9 | 1.1 | 0.7 | 1.3 | 1.3 |
| **mRNA expression** | 6 | 2.4 | 2.2 | 1.6 | 1.4 | 1.9 | 3.3 |
|  | 24 | 1.5 | 108.7 | 2.1 | 0.6 | 5.0 | 23.4 |
|  | 48 | 20.0 | 15.7 | 4.0 | 7.8 | 13.0 | 14.5 |
|  | 72 | 26.9 | 17.3 | 22.9 | 33.1 | 12.3 | 33.6 |
| **Phosphorylation of** | M | 1.0 | 1.0 | 1.0 | 1.0 | 1.0 | 1.0 |
| **STAT2** | 1 | 0.1 | 0.1 | 0.1 | 0.9 | 0.4 | 1.4 |
|  | 6 | 0.0 | 0.1 | 0.1 | 1.0 | 0.7 | 1.5 |
|  | 24 | 8.2 | 25.7 | 0.1 | 7.1 | 30.2 | 20.2 |
|  | 48 | 19.7 | 13.6 | 7.0 | 14.5 | 8.9 | 10.8 |
|  | 72 | 4.4 | 8.4 | 4.3 | 9.2 | 4.6 | 9.8 |

**Supplementary Table 1. Summary of various SARS-CoV-2 variants replication in Calu-3 cells, host cell interferon expression and phosphorylation levels of STAT2.** The table lists the relative cellular vRNA levels, relative mRNA expression levels of IFN-β1, IFN-λ1, IFN-λ2 and CXCL10 and the relative phosphorylation of STAT2 normalized to the mock sample. The highest values for each variant are highlighted in yellow.

|  | **h p.i.** | **Fin55-BA.1** | **Fin58-BA.2** | **Fin60-XJ** | **Fin34-α** | **Fin37-δ** |
| --- | --- | --- | --- | --- | --- | --- |
| **Replication** | 1 | 4.03E+02 | 2.34E+03 | 1.30E+03 | 6.91E+02 | 3.90E+02 |
| **Relative cellular** | 6 | 2.14E+02 | 1.54E+03 | 8.31E+02 | 1.62E+03 | 3.67E+03 |
| **vRNA expression** | 24 | 6.70E+04 | 4.89E+04 | 2.00E+05 | 1.03E+06 | 3.73E+06 |
|  | 48 | 7.43E+05 | 7.35E+04 | 2.40E+06 | 6.94E+06 | 5.98E+06 |
|  | 72 | 6.86E+05 | 5.47E+04 | 1.95E+06 | 2.73E+06 | 4.42E+06 |
| **Relative IFN-β1** | 1 | 0.6 | 0.6 | 1.6 | 0.5 | 1.3 |
| **mRNA expression** | 6 | 1.1 | 2.7 | 0.9 | 2.6 | 2.2 |
|  | 24 | 6.1 | 14.3 | 13.2 | 19.7 | 204.0 |
|  | 48 | 165.7 | 17.8 | 347.2 | 729.8 | 1605.3 |
|  | 72 | 396.3 | 22.1 | 450.5 | 542.5 | 544.0 |
| **Relative IFN-λ1** | 1 | 0.4 | 0.6 | 0.8 | 0.4 | 0.8 |
| **mRNA expression** | 6 | 0.6 | 1.1 | 0.8 | 1.4 | 1.8 |
|  | 24 | 1.5 | 2.8 | 4.0 | 6.3 | 96.9 |
|  | 48 | 87.7 | 7.4 | 192.6 | 377.3 | 418.4 |
|  | 72 | 138.5 | 17.2 | 319.6 | 167.1 | 210.8 |
| **Relative IFN-λ2** | 1 | 0.3 | 0.8 | 0.9 | 0.2 | 1.5 |
| **mRNA expression** | 6 | 0.4 | 2.4 | 0.8 | 1.2 | 2.3 |
|  | 24 | 2.7 | 5.1 | 8.8 | 13.0 | 234.1 |
|  | 48 | 193.4 | 16.6 | 433.1 | 744.6 | 606.3 |
|  | 72 | 330.2 | 25.8 | 578.3 | 279.0 | 220.1 |
| **Relative CXCL10** | 1 | 0.7 | 0.5 | 1.0 | 0.3 | 0.8 |
| **mRNA expression** | 6 | 0.8 | 1.1 | 0.8 | 1.2 | 1.5 |
|  | 24 | 0.4 | 1.0 | 1.5 | 1.5 | 20.0 |
|  | 48 | 7.0 | 1.7 | 15.5 | 15.2 | 21.7 |
|  | 72 | 6.5 | 2.4 | 18.4 | 9.0 | 13.4 |

**Supplementary Table 2. Summary of the viral replication level in Calu-3 cells and host cell interferon expression of various Omicron sublineages compared to Fin34-α and Fin37-δ.** The table lists the relative cellular vRNA levels and relative mRNA expression levels of IFN-β1, IFN-λ1, IFN-λ2 and CXCL10. The highest values for each sublineage and variant are highlighted in yellow.
